# Neutrophils are important for the development of pro-reparative macrophages after irreversible electroporation of the liver in mice

**DOI:** 10.1038/s41598-021-94016-8

**Published:** 2021-07-22

**Authors:** Maya Lopez-Ichikawa, Ngan K. Vu, Amar Nijagal, Boris Rubinsky, Tammy T. Chang

**Affiliations:** 1 Department of Surgery, University of California, San Francisco, 513 Parnassus Avenue, San Francisco, CA 94143 USA; 2 Department of Mechanical Engineering, University of California, Berkeley, 6124 Etcheverry Hall, Berkeley, CA 94720 USA

**Keywords:** Liver, Innate immune cells

## Abstract

Irreversible electroporation (IRE) is a non-thermal tissue ablative technology that has emerging applications in surgical oncology and regenerative surgery. To advance its therapeutic usefulness, it is important to understand the mechanisms through which IRE induces cell death and the role of the innate immune system in mediating subsequent regenerative repair. Through intravital imaging of the liver in mice, we show that IRE produces distinctive tissue injury features, including delayed yet robust recruitment of neutrophils, consistent with programmed necrosis. IRE treatment converts the monocyte/macrophage balance from pro-inflammatory to pro-reparative populations, and depletion of neutrophils inhibits this conversion. Reduced generation of pro-reparative Ly6C^lo^F4/80^hi^ macrophages correlates with lower numbers of SOX9^+^ hepatic progenitor cells in areas of macrophage clusters within the IRE injury zone. Our findings suggest that neutrophils play an important role in promoting the development of pro-reparative Ly6C^lo^ monocytes/macrophages at the site of IRE injury, thus establishing conditions of regenerative repair.

## Introduction

Irreversible electroporation (IRE) consists of short, high electric field pulses that induce permanent nanopores in cell membranes, causing loss of cellular homeostatic control and cell death^1^. The mode of cell killing is non-thermal; therefore, the surrounding extracellular matrix is not denatured and remains intact. This feature is likely important for the ability of IRE-ablated tissues, including normal skin^2^, intestine^3^, and liver^4–6^, to regenerate with minimal formation of scar. Because of these non-thermal and extracellular matrix-preserving properties, IRE has become a valuable clinical modality to ablate tumors near critical structures, such as blood vessels, ducts, and nerves^7–10^. Furthermore, IRE may be used to decellularize organs, either ex vivo or in vivo, to advance tissue engineering and cellular transplantation objectives^11–13^.

It is not completely understood how dead cells are cleared and subsequently replaced by new cells after IRE. The mechanism through which IRE induces cell death is controversial. Several studies suggest that IRE kills cells by apoptosis^14–16^. There is also evidence that IRE activates other forms of cell death, including necrosis, necroptosis, and pyroptosis^17–19^. Apoptosis is noninflammatory cell death, whereas necrosis, necroptosis, and pyroptosis are inflammatory^20,21^. Apoptotic cells release short-range signals that stimulate local resident phagocytes to engulf them^22^, and simultaneously produce factors to inhibit neutrophil recruitment^23^. In contrast, inflammatory cell death leads to the release of damage-associated molecular patterns that activate neutrophils, initiating the cascade of circulating monocyte recruitment and engagement of the broader immune system^21^. Therefore, characterizing the neutrophil response after IRE tissue ablation would provide significant insight as to whether apoptosis or other inflammatory forms of cell death predominate.

Moreover, understanding the effect of IRE on inflammatory and resolution pathways is critical to our ability to use IRE as a tool to improve human health. On the one hand, maximizing immunogenic cell death is likely beneficial for oncologic applications of IRE. There is evidence that IRE increases the efficacy of immunotherapy in treating cancers that are resistant to immune checkpoint inhibitors^24^. On the other hand, minimizing deleterious inflammation is advantageous for IRE-mediated in vivo organ decellularization to facilitate engraftment of stem cell-derived cells for regenerative surgery^13^.

We hypothesized that IRE induces distinctive qualities of neutrophil activation, which play important roles in establishing the ensuing favorable milieu for regenerative tissue repair. To test our hypothesis, we performed detailed intravital imaging analysis of neutrophil trafficking after IRE treatment of liver parenchyma. We characterized the neutrophil and monocyte/macrophage responses by multi-color flow cytometry. Finally, we depleted neutrophils by antibody administration and determined the effect on post-IRE tissue repair.

## Results

### IRE induces distinctive tissue injury and neutrophil recruitment features

To track tissue changes and neutrophil behavior at very early timepoints after IRE, we performed intravital imaging on LysM-eGFP mice immediately after IRE was applied to the liver. Neutrophils were identified as eGFP^+^ cells, as confirmed by staining with neutrophil-specific anti-Ly6G-PE antibody given intravenously. Vasculature and cell death in the liver parenchyma were stained by intravenous administration of antibodies to platelet endothelial cell adhesion molecule-1 (anti-PECAM-1-PE) and SYTOX Blue, respectively.

**Figure 1:**
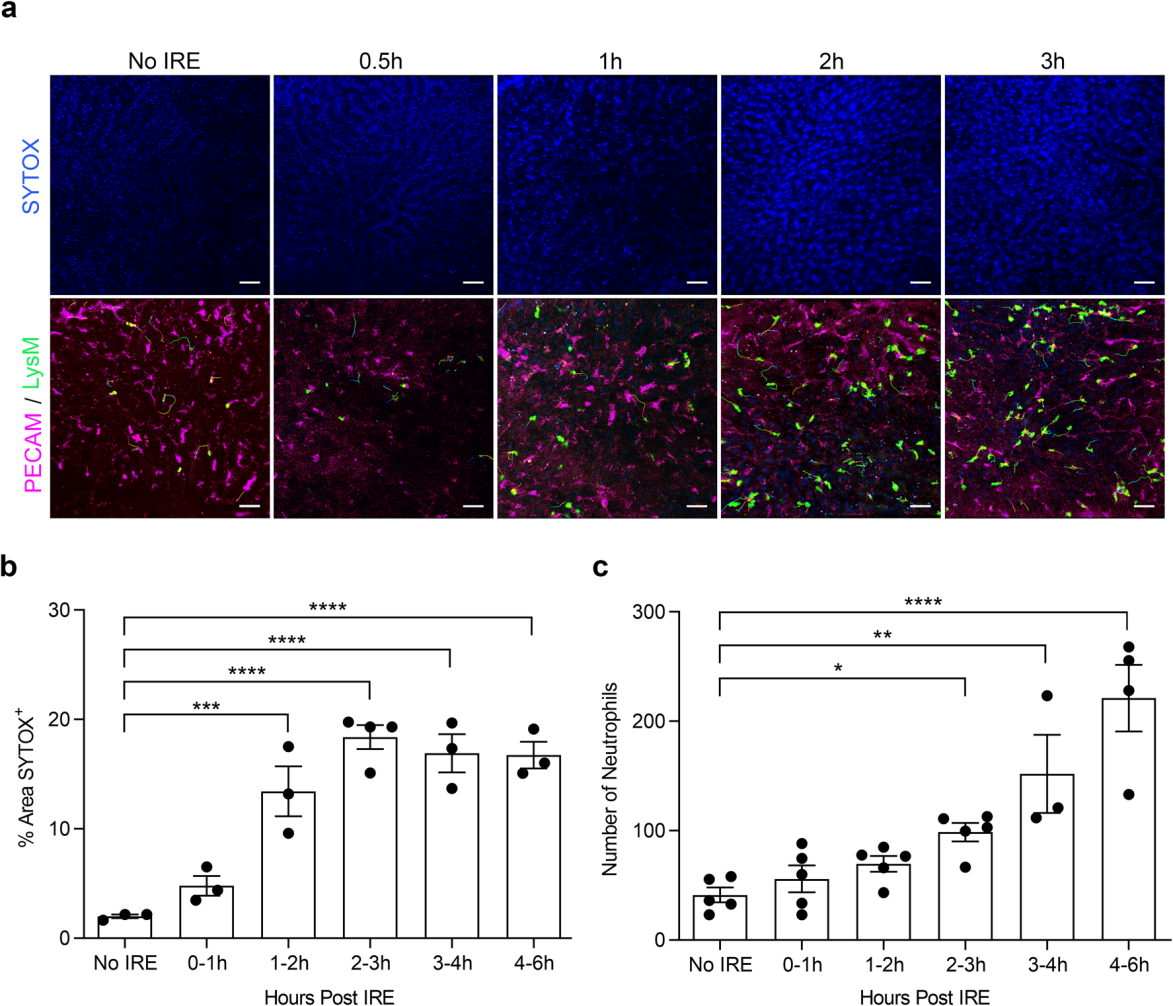
Intravital imaging demonstrates patent microvasculature, delayed cell death, and neutrophil recruitment after IRE. (**a**) LysM-eGFP mice, in which neutrophils express eGFP, were given intravenous anti-PECAM-1-PE to label endothelial cells and SYTOX Blue to identify dead cells. Intravital imaging of the liver was performed without IRE or at early timepoints after 1500 V/cm IRE treatment. (**b**) Percentage of liver parenchyma area positive for SYTOX per intravital image field (500μmx520μm field of view with 25 × objective) was determined at baseline (No IRE) and at the indicated time spans after IRE treatment. (**c**) Numbers of neutrophils (eGFP^+^ cells) per intravital image field were counted at baseline (No IRE) and at the indicated time spans after IRE treatment. For (**b**,**c**), one-way ANOVA shows significant differences between No IRE and all of the time brackets after IRE (*p* < 0.0001). Post hoc multiple comparison with Holm–Sidak correction of each time bracket to the No IRE baseline demonstrates *p < 0.05, **p < 0.01, ***p < 0.001, and ****p < 0.0001. Individual points represent independent animals (n = 3–5 per condition). Graph bars and error bars show mean ± SEM.

We found several important differences in the immediate tissue response to IRE-mediated injury as compared to thermal-mediated injury reported previously in the literature^25,26^. After a focal 0.02mm^3^ burn to the liver surface, there is immediate lytic necrotic cell death as demonstrated by the appearance of propidium iodide-positive cells at the injury site within minutes^25^. In contrast, after IRE ablation of approximately 4700–5700mm^3^ of liver tissue (10 mm-diameter clamp electrodes applied to the full-thickness of a liver lobe ranging 15-18 mm in thickness), the kinetics of cell death were much slower, and SYTOX^+^ nuclear staining increased significantly 1-2 h after IRE (Fig. 1a,b). After thermal insult, microvasculature collapsed at the site of injury^25,26^. Neutrophils accumulated at the periphery of the injury site and then dismantled the collapsed microvessels in order to infiltrate the injury area and clear cellular debris^25,26^. Conversely, microvasculature within IRE-injured tissues remained patent (Fig. 1a and Supplemental Movies 1–5). PECAM-1 staining of sinusoidal endothelium remained bright and contiguous after IRE treatment, and neutrophils coursed through patent PECAM-1^+^ sinusoids deep within the IRE injury zone. Intravenous Evans Blue dye showed continuous blood flow through sinusoids in tissues treated with IRE, and neutrophils traveled freely through these vessels (Supplemental Movie 6). Finally, whereas thermal injury induced rapid recruitment of neutrophils to the site within 30–60 minutes^25,26^, recruitment of neutrophils in response to IRE was much slower; neutrophils significantly increased in number 2-3 h after IRE ablation (Fig. 1a,c). Therefore, although the volume of ablated tissue in our IRE model was much larger than in the published thermal injury model, the recruitment of neutrophils to the injury site was slower after IRE than compared to thermal injury. Moreover, the behavior of recruited neutrophils differed between the 2 models. Unlike after thermal injury, in which neutrophils first surrounded and then infiltrated the injury site, neutrophils recruited after IRE trafficked throughout the injury zone via patent microvessels. These observations demonstrate distinctive early features of IRE-induced tissue injury and neutrophil response.

### Neutrophil movement changes dynamically in the first few hours after IRE and neutrophil numbers remain elevated in the injury zone for up to 3 days

**Figure 2:**
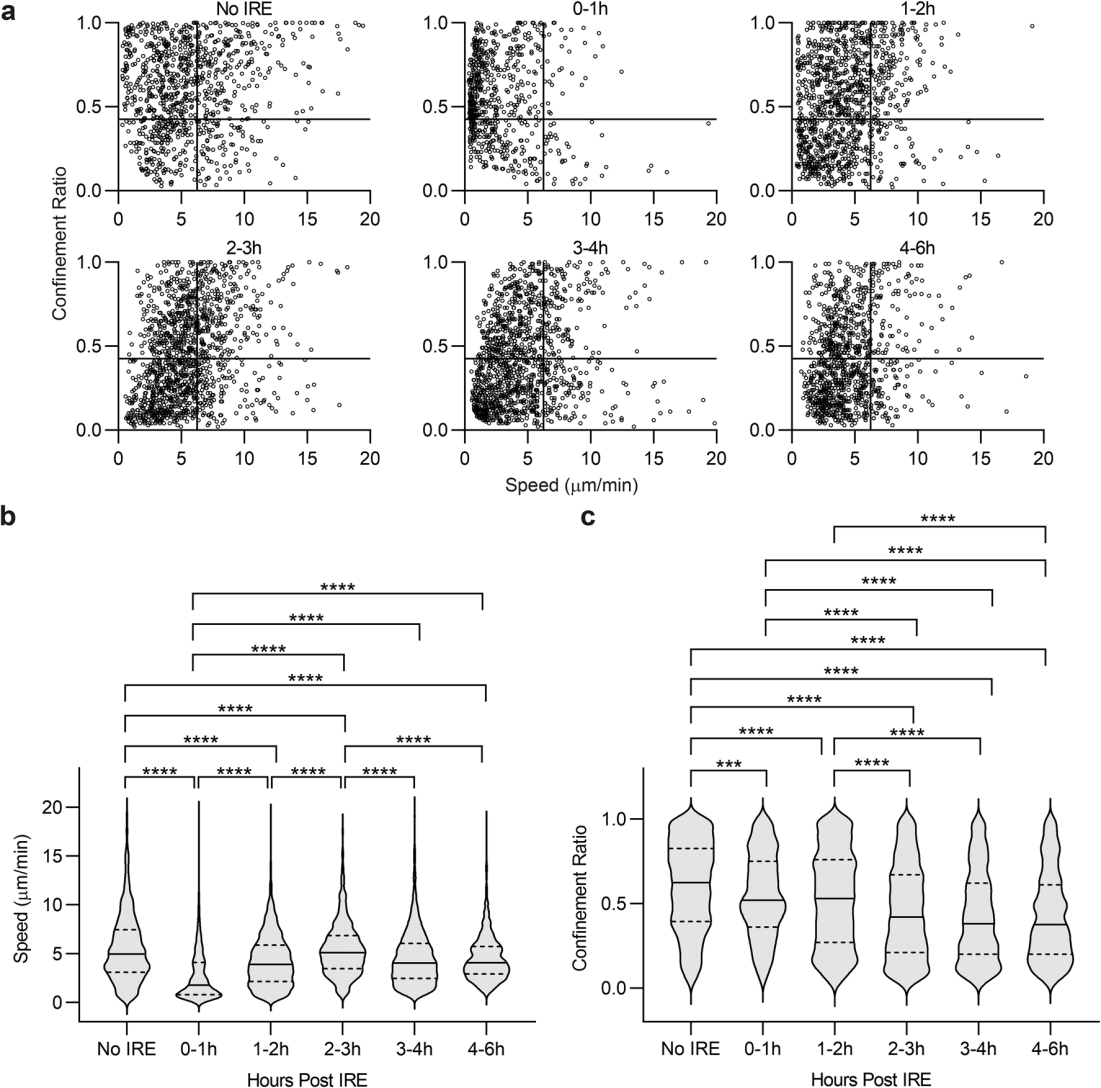
Neutrophil speed and confinement ratio change dynamically after IRE. (**a**) Speed and confinement ratio (displacement/track length) of neutrophils were determined by intravital imaging in liver parenchyma at baseline (No IRE) and for each hour after 1500 V/cm IRE treatment up to the 4-6 h time bracket. Each point on the scatter graphs represents a single cell, with its speed plotted against its confinement ratio. Each graph shows the movement characteristics of 680–1290 individual neutrophils, pooled from 4–6 independent experiments using separate animals. (**b**) Speed and (**c**) Confinement ratio of tracked neutrophils are represented in violin plots, in which solid lines show medians and dotted lines show quartiles. For both parameters, one-way ANOVA indicates significant differences between No IRE and all time brackets after IRE (*p* < 0.0001). Post hoc Tukey multiple comparison demonstrates ***p < 0.001 and ****p < 0.0001.

Speed and confinement ratio (displacement / total track length) in combination characterize leukocyte activation states^27–29^. Moreover, decreasing speed and confinement ratio are associated with neutrophil effector functions^30,31^. We analyzed the migration speeds and confinement ratios of neutrophils at early timepoints after IRE and found that both were highly dynamic in the first few hours. At baseline, neutrophils passing through liver sinusoids exhibited a range of speeds and confinement ratios (Fig. 2a). Neutrophil speed in the injury zone slowed down markedly within the first hour after IRE (Fig. 2a,b), increased in the next 2 hours, and gradually slowed again around 3 h after IRE. Similarly, confinement ratios decreased significantly after IRE within the first hour, returned to baseline levels briefly, and then decreased to a new steady state 2–3 h after IRE (Fig. 2a,c). These results suggest that there may be 2 phases to the initial neutrophil response after IRE. The first phase, within an hour after injury, may represent an immediate response of “bystander” neutrophils traveling through the liver at the time of injury. The second phase, 2–3 h later, in which neutrophils exhibited sustained lower speeds and confinement ratios, and which corresponded to when neutrophil numbers significantly increased (Fig. 1c), may represent neutrophils actively recruited to the site in response to the injury.

**Figure 3:**
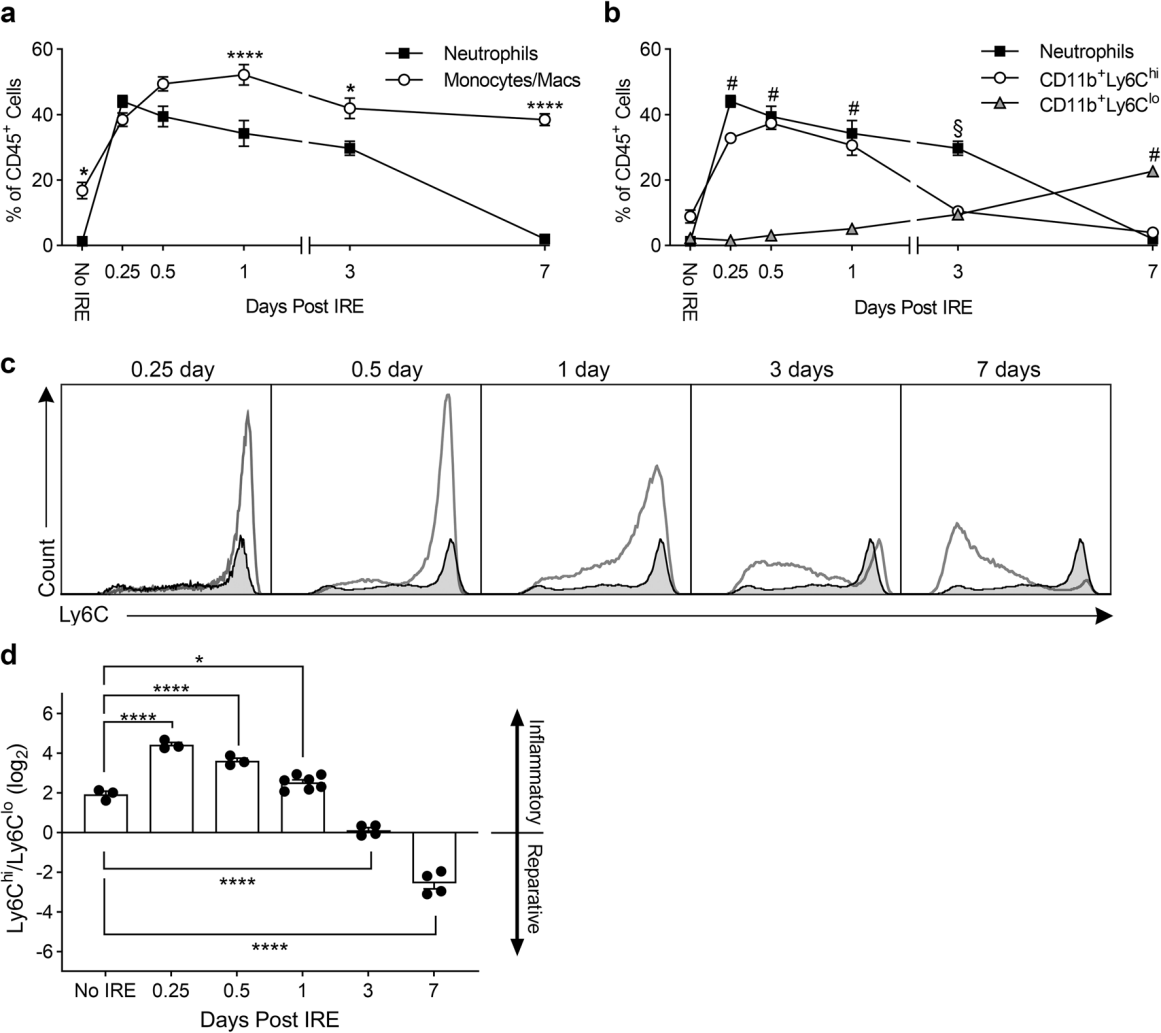
Neutrophils and CD11b^+^Ly6C^hi^ monocytes/macrophages predominate early after IRE, whereas CD11b^+^Ly6C^lo^ monocytes/macrophages predominate later. (**a**) Myeloid cells were isolated from control (No IRE) or IRE-treated liver, and multi-color flow cytometry determined the numbers of neutrophils and monocytes/macrophages through time. (**b**) CD11b^+^ monocytes/macrophages were subdivided into Ly6C^hi^ and LyC^lo^ populations to show the relationship with neutrophils. For (**a**,**b**), cell type and time were assigned as independent variables, and 2-way ANOVA showed significant interaction between the two factors (*p* < 0.0001). In (**a**), Holm–Sidak multiple comparison showed significant differences between neutrophils and monocytes/macrophages in livers that received No IRE and in livers 1, 3, and 7 days after IRE (*p < 0.05, ****p < 0.0001). In (**b**), Tukey multiple comparison showed significant differences ^#^between neutrophils and CD11b^+^Ly6C^hi^ compared to CD11b^+^Ly6C^lo^ on days 0.25, 0.5, 1, and 7, and ^§^between neutrophils compared to CD11b^+^Ly6C^hi^ and CD11b^+^Ly6C^lo^ on day 3 (*p* < 0.0001). Data represent mean ± SEM with 3–7 independent biological replicates (n = 3–7). (**c**) Representative flow cytometry histograms of Ly6C expression on CD11b^+^ monocytes/macrophages after IRE treatment. Shaded histograms represent Ly6C expression on CD11b^+^ monocytes/macrophages in control liver that did not undergo IRE. (**d**) Ly6C^hi^ to Ly6C^lo^ monocyte/macrophage ratio in control liver (No IRE) and in liver treated with IRE through time. One-way ANOVA indicate significant differences between No IRE and timepoints after IRE treatment (*p* < 0.0001). Holm–Sidak multiple comparison comparing No IRE to each of the post-IRE timepoints shows *p < 0.05 and ****p < 0.0001 as indicated. Individual points represent independent biological replicates (n = 3–7). Graph bars and error bars show mean ± SEM.

To track the neutrophil response at later timepoints and to characterize the interaction between neutrophils and monocytes/macrophages, we isolated liver myeloid cells and performed multi-color flow cytometry. (See Supplemental Fig. 1 for gating strategy and definition of subpopulations.) At baseline prior to IRE ablation, neutrophils comprised only 1% of all CD45^+^ leukocytes in the liver (Fig. 3a). After IRE, consistent with accelerating neutrophil accumulation starting at 2-3 h determined by intravital imaging (Fig. 1c), neutrophil numbers within the injury area peaked by 6–12 h as measured by flow cytometry (Fig. 3a). Interestingly, neutrophils remained a major population at the IRE injury site for at least 3 days, constituting 30–45% of CD45^+^ leukocytes. Neutrophil populations eventually decreased back to baseline levels by day 7 after IRE (Fig. 3a). This prolonged presence of neutrophils after IRE is different from the focal thermal injury model, in which the number of neutrophils peaks at 12 h and then rapidly declines by 24 h^25,26^.

### IRE converts liver monocyte/macrophage populations from pro-inflammatory to pro-reparative

As opposed to neutrophils that constitute a very small percentage (1%) of CD45^+^ leukocytes in the liver at baseline, CD11b^+^ monocytes/macrophages are a significant leukocyte population in the normal liver and represent nearly 20% of CD45^+^ liver leukocytes (Fig. 3a). After IRE, monocyte/macrophage numbers increased concurrently with neutrophils and peaked at day 1. However, unlike neutrophils, CD11b^+^ monocyte/macrophage numbers remained persistently high (> 40% of CD45^+^ leukocytes) through day 7 (Fig. 3a).

Liver macrophage populations are highly complex and heterogenous^32,33^. Bone-marrow-derived monocytes/macrophages in the liver may be classified as CD11b^+^Ly6C^hi^, corresponding to a pro-inflammatory phenotype, or CD11b^+^Ly6C^lo^, which has pro-reparative functions^32,34^. Determining the composition of monocyte/macrophage subsets revealed that pro-inflammatory CD11b^+^Ly6C^hi^ cells predominated in the first 24 h and significantly decreased by 3 days after IRE. On the other hand, CD11b^+^Ly6C^lo^ cells gradually increased over time and became the dominant population by day 7 (Fig. 3b,c). The ratio of CD11b^+^Ly6C^hi^ to CD11b^+^Ly6C^lo^ monocytes/macrophages suggests that the innate immunity cell populations in the liver was pro-inflammatory at baseline and that the pro-inflammatory state was further augmented in the first 24 h after IRE (Fig. 3d). The balance is then tipped toward CD11b^+^Ly6C^lo^ cells with an inflection point at day 3, transitioning to a primarily pro-reparative population by day 7.

### CD11b^+^Ly6C^lo^ cells that predominate in the liver after IRE treatment display markers of activated macrophages

**Figure 4:**
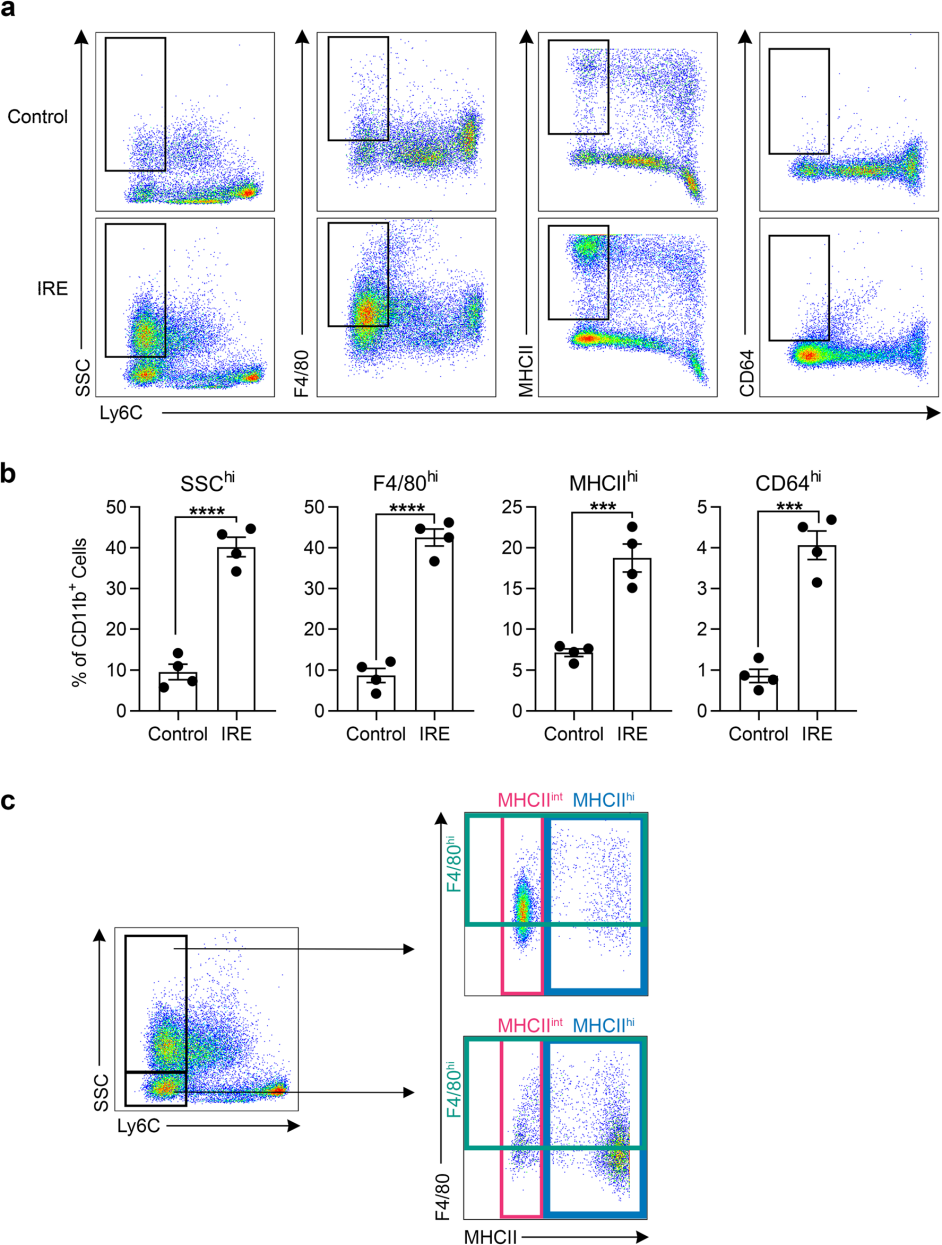
The majority of CD11b^+^Ly6C^lo^ cells that develop in the liver after IRE treatment are side-scatter high, and express high levels of F4/80 and intermediate levels of MHCII. (**a**) Representative flow cytometry density plots showing Ly6C expression versus side-scatter (SSC), F4/80, MHCII, and CD64 of CD11b^+^ monocytes/macrophages isolated from the IRE-treated liver lobe compared to parts of the liver not directly treated by IRE (control) from the same animal. Rectangular gates define Ly6C^lo^ cells that are “high” for the given marker. (**b**) Quantification of CD11b^+^Ly6C^lo^ cells that are SSC, F4/80, MHCII, or CD64 high, as defined by the gates in (**a**), in control and IRE-treated parts of the liver. Two-tailed unpaired t tests show ***p < 0.001 and ****p < 0.0001. Individual points represent data from separate animals (n = 4). Graph bars and error bars show mean ± SEM. (**c**) Representative flow cytometry density plots showing that the majority (70%) of CD11b^+^Ly6C^lo^ cells in IRE-treated liver are SSC^hi^, and that this subset is also F4/80^hi^ and MHCII^int^. The remainder of the CD11b^+^Ly6C^lo^ cells that are SSC^lo^ (30%) express lower levels of F4/80 and very high levels of MHCII.

We further analyzed the phenotype of the monocytes/macrophages that predominated the immune landscape 7 days after IRE treatment. We isolated liver myeloid cells from the IRE treatment area and compared them to myeloid cells isolated from the rest of the liver that did not undergo IRE. We found that significantly higher proportions of CD11b^+^Ly6C^lo^ cells in IRE treatment zones were side-scatter (SSC) high and F4/80 high, corresponding to a macrophage phenotype (Fig. 4a,b). Additionally, significantly more CD11b^+^Ly6C^lo^ cells in IRE zones expressed high levels of major histocompatibility complex II (MHCII) and Fc-gamma receptor I (CD64), consistent with antigen-presenting functions. Within the CD11b^+^Ly6C^lo^ population, 70% of the cells were SSC^hi^ and the remainder were SSC^lo^ (Fig. 4c). Further analysis showed that Ly6C^lo^SSC^hi^ cells expressed high F4/80 and intermediate MHCII, whereas Ly6C^lo^SSC^lo^ cells expressed lower levels of F4/80 and high MCHII, indicating 2 subsets within the CD11b^+^Ly6C^lo^ population. These results indicate that 7 days after IRE treatment, CD11b^+^Ly6C^lo^ cells within injury zones display phenotypic markers consistent with differentiation toward activated macrophages.

### Neutrophils are important for generating pro-reparative CD11b^+^Ly6C^lo^ macrophages in the liver after IRE treatment

**Figure 5:**
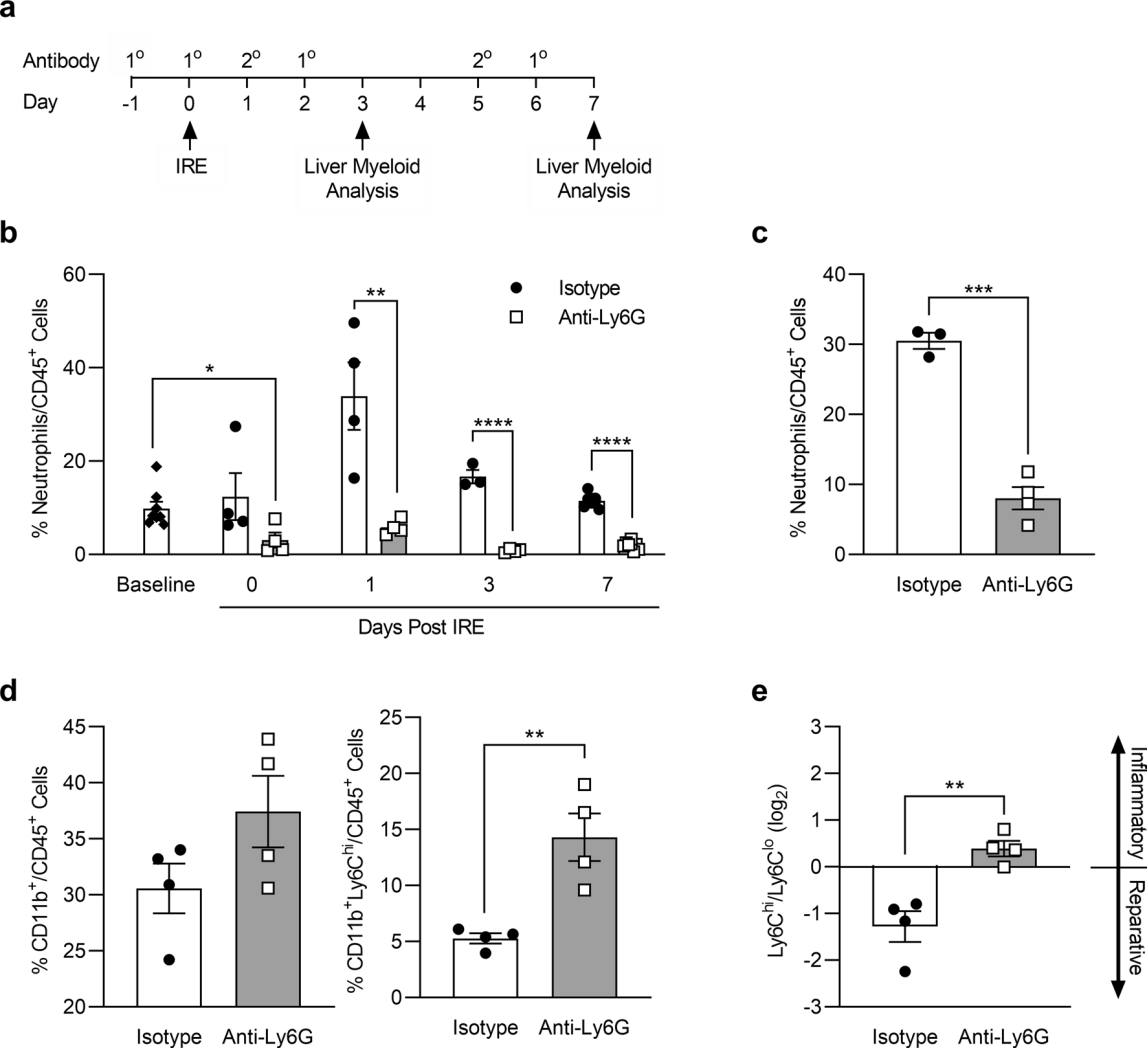
Neutrophil depletion increases the proportion of CD11b^+^Ly6C^hi^ cells in the liver after IRE and shifts the macrophage population toward a more pro-inflammatory state. (**a**) Neutrophil depletion protocol and timeline in which rat IgG2a (isotype control) or anti-Ly6G were given as 1° antibodies and anti-rat IgG given as the cross-linking 2° antibody. The timing of IRE treatment and analysis of liver myeloid cells are indicated by arrows. (**b**) Percentage of neutrophils in the blood at baseline (prior to any IRE or antibody treatments), and on days 0, 1, 3, and 7 after IRE treatment with the neutrophil depletion regimen shown in (**a**). (**c**) Percentage of neutrophils in IRE-treated liver parenchyma 3 days after treatment in mice given isotype control or anti-Ly6G. (**d**) Total CD11b^+^ cells or CD11b^+^Ly6C^hi^ cells as a percentage of CD45^+^ leukocytes in IRE-treated liver parenchyma 7 days after treatment in mice with or without neutrophil depletion. (**e**) Ly6C^hi^ to Ly6C^lo^ ratio in IRE-treated liver 7 days after treatment in mice given isotype control versus anti-Ly6G. For all graphs, unpaired two-tailed t tests show *p < 0.05, **p < 0.01, ***p < 0.001, and ****p < 0.0001. Data points represent individual animals in each condition and/or timepoint (n = 3–8). Graph bars and error bars show mean ± SEM.

To determine the role neutrophils may play in the development of CD11b^+^Ly6C^lo^ pro-reparative macrophages after IRE, we depleted neutrophils by administering anti-Ly6G antibody and cross-linking secondary antibody (Fig. 5a)^35^ and analyzed the liver myeloid populations by flow cytometry. (See Supplemental Fig. 2 for gating strategy). Anti-Ly6G antibody-mediated depletion significantly reduced circulating levels of neutrophils in the blood prior to the application of IRE and throughout the 7-day period after IRE treatment, as compared to administration of isotype control as the primary antibody (Fig. 5b). Neutrophil numbers in the livers of mice treated with anti-Ly6G after IRE were significantly reduced, indicating that depletion of neutrophils in the blood resulted in significantly reduced recruitment of neutrophils to the liver (Fig. 5c). Neutrophil depletion led to significantly increased numbers of CD11b^+^Ly6C^hi^ cells in the liver 7 days after IRE, as compared to isotype controls (Fig. 5d). In contrast to control conditions in which the Ly6C^hi^/Ly6C^lo^ balance favored a pro-reparative population, neutrophil depletion switched the Ly6C^hi^/Ly6C^lo^ balance toward a pro-inflammatory state (Fig. 5e). These findings demonstrate that neutrophils play an important role in promoting the establishment of pro-reparative CD11b^+^Ly6C^lo^ macrophages after IRE.

### Neutrophil depletion increases the proportion of pro-inflammatory macrophages that may interfere with post-IRE liver repair and regeneration

**Figure 6:**
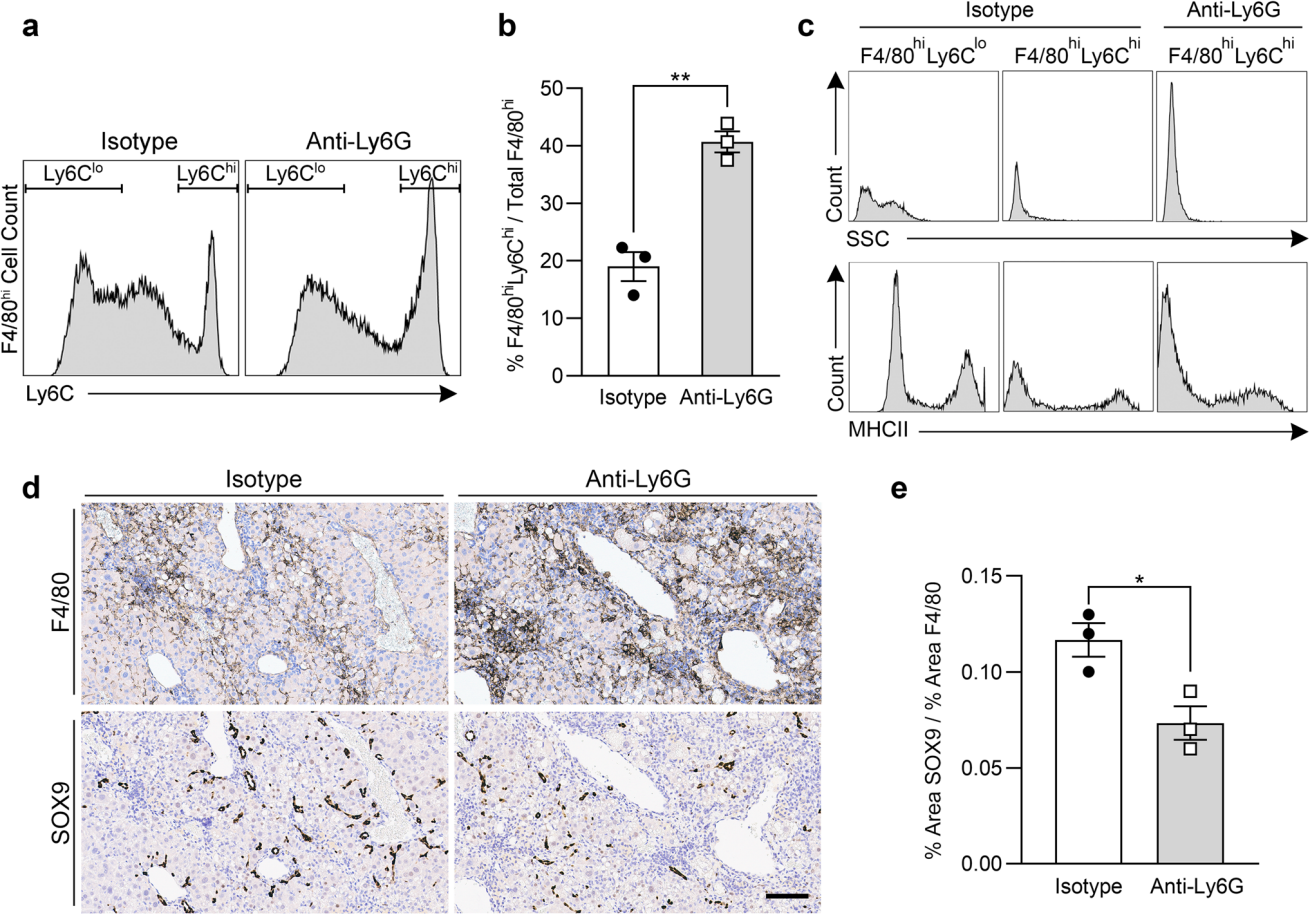
Neutrophil depletion increases the proportion of F4/80^hi^Ly6C^hi^ macrophages in IRE-treated liver, and areas of F4/80^+^ macrophage clusters correlate with lower numbers of SOX9^+^ liver progenitors. (**a**) Representative flow cytometry histograms showing Ly6C expression in CD11b^+^F4/80^hi^ macrophages isolated from IRE-treated liver 7 days post treatment in mice given isotype control or anti-Ly6G to deplete neutrophils. Gates show subpopulations defined as Ly6C^hi^ or Ly6C^lo^. (**b**) F4/80^hi^Ly6C^hi^ macrophages as a percentage of total F4/80^hi^ macrophages in IRE-treated liver parenchyma of mice with or without neutrophil depletion. (**c**) Representative histograms comparing the SSC and MHCII characteristics of F4/80^hi^Ly6C^hi^ macrophages in neutrophil-depleted mice with the macrophage subsets in control mice. (**d**) Representative immunohistochemistry images of F4/80 and SOX9 expression in IRE-treated liver 7 days after treatment in control and neutrophil-depleted mice (scale bar = 100 μm). (**e**) Quantification of SOX9 staining relative to F4/80 staining in the liver of isotype and anti-Ly6G-treated mice 7 days post IRE. Representative histograms and histology images were chosen from among data presented in (**b**,**e**), in which unpaired two-tailed t tests show *p < 0.05 and **p < 0.01. Data points represent individual animals (n = 3). Graph bars and error bars show mean ± SEM.

We further analyzed the F4/80^hi^ macrophage population that developed in neutrophil-depleted mice. At 7 days post-IRE, the proportion of F4/80^hi^Ly6C^hi^ macrophages was significantly increased in neutrophil-depleted mice as compared to controls (Fig. 6a,b). Whereas the F4/80^hi^Ly6C^lo^ macrophages that predominated in control mice were SSC^hi^ and MHCII^int/hi^, the F4/80^hi^Ly6C^hi^ macrophages that developed in neutrophil-depleted mice were SSC^lo^ and MHCII^lo/int^ (Fig. 6c). Immunohistochemistry of the liver parenchyma showed denser clusters of F4/80^+^ macrophages in neutrophil-depleted mice as compared to control mice (Fig. 6d). SRY-related HMG box transcription factor 9-positive (SOX9^+^) progenitor cells constitute one mechanism through which the liver regenerates after IRE injury^5^. In neutrophil-depleted mice, SOX9^+^ progenitor cells were sparse in areas dominated by F4/80^+^ clusters (Fig. 6d), and the ratio of SOX9 to F4/80 tissue staining was significantly lower compared to controls (Fig. 6e). These results suggest that neutrophil depletion increases the proportion of F4/80^hi^Ly6C^hi^ macrophages after IRE, correlating with inhibited and/or delayed induction of SOX9^+^ progenitor cells involved in liver regeneration.

## Discussion

In this study, we found that, unlike what occured after thermal injury^25,26^, microvessels remained patent after IRE and the time-course of cell death was delayed. Although significant accumulation of neutrophils occurred later at 2–3 h, the movement of neutrophils passing through the injury zone was immediately impacted after the application of IRE. Because microvessels remained patent, neutrophils continued to migrate intravascularly and accumulated deep within the IRE injury zone, as opposed in thermal injury, where neutrophils first surrounded the necrotic core and then infiltrated from the periphery toward the center^25,26,36^. Whereas in thermal injury neutrophil numbers rapidly declined by 24 h^25^, neutrophils persisted at the site of IRE injury much longer and remained a major myeloid population for the first 3 days. When neutrophil numbers finally returned to baseline levels at 7 days post-IRE, CD11b^+^Ly6C^lo^ monocytes/macrophages became the dominant myeloid cell population within the injury zone. We showed that IRE treatment shifted the CD11b^+^Ly6C^hi^ versus CD11b^+^Ly6C^lo^ monocyte/macrophage balance from one that was pro-inflammatory at baseline, toward one predominated by pro-reparative CD11b^+^SSC^hi^F4/80^hi^MHC^int^Ly6C^lo^ macrophages by day 7. Neutrophil depletion prevented the transition toward a pro-reparative balance and inhibited the development of F4/80^hi^Ly6C^lo^ macrophages. Increased proportion of F4/80^hi^Ly6C^hi^ macrophages at the IRE injury site in neutrophil-depleted mice correlated with fewer SOX9^+^ hepatic progenitor cells in areas occupied by clusters of F4/80^+^ macrophages.

Because the injury is focal and the precise timing of the tissue insult is known, thermal and IRE are useful models for investigating the dynamics of immune cell recruitment to sites of cell death caused by sterile injury^21^. Apoptotic cell death, through the activation of caspase-3, is immunologically silent, activating only local resident phagocytes for clearance of apoptotic bodies and proactively inhibiting neutrophil recruitment^22,23^. Our data showing robust recruitment of neutrophils after IRE indicate that apoptosis is likely not the dominant pathway of IRE-induced cell death. IRE-mediated cell death is also unlike the necrotic cell death that occurs after thermal injury, in which there is immediate uncontrolled lysis of cells with release of cellular contents into the environment^25,26^. After IRE, the numbers of dead cells, as indicated by SYTOX^+^ nuclei, gradually increase within the injury zone and become significantly greater than non-treated baseline at 1–2 h after injury. This slower time-course of cell death is aligned with the activation of regulated cell death pathways. Our data supports previous observations showing that IRE activates programmed necrosis with upregulated expression of molecular markers of necroptosis (receptor-interacting kinase 3 and mixed lineage kinase domain-like protein) and pyroptosis (cleaved caspase-1 and cleaved gasdermin D) in liver parenchyma treated with IRE^19^. Furthermore, in vitro studies indicate that it takes 1.5–2.5 h from the onset of necroptotic injury to when cells demonstrate SYTOX^+^ nuclei^37^, consistent with the timing of SYTOX^+^ nuclei appearance in vivo after IRE, as we demonstrated by intravital imaging. It remains possible that multiple mechanisms of cell death coexist within a single IRE-ablated zone, and that the specific cell death subroutine each cell undergoes is dependent on multiple factors, including the electroporation parameters, cell type, and local microenvironment^20^. Interestingly, electrochemotherapy, a local anti-cancer treatment that combines electroporation and administration of chemotherapeutic drugs, has been shown to induce immunogenic cell death, likely necroptosis, which is important for generating a systemic anti-tumor response^38,39^.

In addition to focal thermal injury^36^, other models of sterile liver injury, including carbon tetrachloride-induced fibrosis^34^ and acute acetaminophen toxicity^40^, have shown that pro-inflammatory monocytes (defined as Ly6C^hi^, Ly6C^hi^CX_3_CR1^lo^, or CCR2^hi^CX_3_CR1^lo^) are initially recruited, followed by in situ conversion to pro-reparative monocytes/macrophages (defined as Ly6C^lo^, Ly6C^lo^CX_3_CR1^hi^, or CCR2^lo^CX_3_CR1^hi^) in the resolution phase. Using adoptive transfer and fluorescent macrophage marker fate-tracing approaches, these studies showed strong evidence that reparative Ly6C^lo^ monocyte/macrophages phenotypically transitioned from recruited inflammatory Ly6C^hi^ monocytes and were not a separately recruited population from the circulation^34,36,40^. Our findings suggest that IRE may be yet another form of sterile liver injury that initially recruited inflammatory CD11b^+^Ly6C^hi^ monocytes, which then gradually converted to CD11b^+^Ly6C^lo^F4/80^hi^ macrophages by day 7. Moreover, we showed that neutrophils were important in mediating the shift from pro-inflammatory toward pro-reparative macrophage populations. Our results are consistent with those in the acetaminophen toxicity model, in which neutrophil depletion inhibited the generation of Ly6C^lo^CX_3_CR1^hi^ macrophages, slowed tissue repair, and reduced hepatocyte regenerative proliferation^40^. Although there is some evidence to suggest that neutrophils are needed to recruit classical pro-inflammatory monocytes^41,42^, our data, and data from others^36,43^, indicate that there are likely parallel pathways for neutrophil and monocyte recruitment, because neutrophil depletion does not significantly decrease monocyte accumulation. Instead, our results support the notion that neutrophils are important in promoting the conversion of pro-inflammatory monocytes into pro-reparative macrophages, thereby facilitating tissue repair and regeneration^40^.

Infiltrating monocytes, and macrophages derived from them, have important and diverse functions in mediating liver homeostasis and disease^32^. In chronic liver injury, such as viral hepatitis, alcoholic liver disease, and nonalcoholic fatty liver disease, recruited Ly6C^hi^ monocytes promote inflammation and fibrosis progression^44–48^. However, when injury is removed, monocyte-derived Ly6C^lo^ macrophages predominate and mediate fibrosis regression by expressing metalloproteinases, deactivating myofibroblasts, and producing anti-inflammatory factors^34,49,50^. In acute liver injury, such as acetaminophen-induced or ischemic-reperfusion injury, monocyte-derived Ly6C^lo^ macrophages have been shown to promote tissue repair and restore sinusoidal structure^40,51–53^. In addition to repair, macrophages are also important in promoting liver regeneration^54^. After partial hepatectomy, liver macrophages and macrophage-derived cytokines, such as tumor necrosis factor alpha and interleukin-6, are critical for promoting restorative hepatocyte proliferation^55–58^. In some forms of liver injury, including IRE^5^, SOX9^+^ bipotential progenitor cells are induced in periportal zones^59–61^. SOX9^+^ progenitors can give rise to hepatocytes and cholangiocytes^62,63^. They form organoids *in vitro*^64,65^ and may respond to Wnt signals^66,67^. We showed that failure to transition toward pro-reparative macrophages in neutrophil-depleted mice correlated with decreased numbers of SOX9^+^ progenitor cells in IRE-injured areas with increased Ly6C^hi^F4/80^hi^ macrophage clusters. These findings suggest that IRE may mediate pro-repair and pro-regenerative effects by preferentially generating Ly6C^lo^ macrophages. The precise mechanisms through which IRE-recruited neutrophils induce Ly6C^lo^ macrophages and promote liver regeneration are topics of interest and future research.

In conclusion, our findings have significant implications for the role of neutrophil activation in clinical applications of IRE. In using IRE for tumor ablations, the recruitment of neutrophils may be important for the generation of Ly6C^lo^MHC^int/hi^ monocytes/macrophages that have high antigen presentation capacity, thereby increasing the display of tumor antigens and overcoming resistance to checkpoint inhibitors^24^. In developing regenerative surgery, IRE-induced neutrophil activation is likely important for promoting the ensuing regenerative milieu that supports exogenous cell engraftment^13^. Continued research into how IRE regulates the innate immune response and tissue repair will advance effective utilization of this technology in improving human health.

## Methods

### Mice

LysM-eGFP^+/−^ mice on a BALB/c background were a gift from Dr. M. Looney (University of California, San Francisco, CA); both male and female mice were used in intravital imaging experiments. Male 6–8-week-old C57BL/6 mice were purchased from Jackson Laboratory (Bar Harbor, ME) and used in flow cytometry and neutrophil depletion experiments. Mice were maintained in a UCSF pathogen-free facility.

### IRE

Anesthesia, aseptic technique, perioperative care, and analgesia were performed in accordance with approved standard procedures and protocols. Anesthesia and analgesia consisted of 2% inhaled isoflurane, 0.05–0.1 mg/kg buprenophine, and 5-10 mg/kg meloxicam. A 1 cm midline incision was made directly below the xiphoid and gentle pressure was applied on both sides of the incision to expose the left lobe of the liver. IRE was performed by gently holding the lateral half of the left lobe between custom 10 mm-diameter circular-plate copper electrodes that were affixed to calipers and connected to an ECM 830 Square Wave Electroporation System (BTX Harvard Apparatus, Holliston, MA). The thickness of the liver lobe of each mouse was measured before IRE administration to determine the voltage required to deliver the prescribed electric field strength. Electrical pulses were applied using parameters of 1500 V/cm, 8 total 100 μs square pulses, each pulse separated by 100 ms. After IRE, the liver lobe was returned to the abdomen, and the abdominal incision was closed by sutures in two layers.

### Intravital microscopy

LysM-eGFP reporter mice received intravenous (IV) injections of 5 μg PECAM-1-PE (390; eBioscience, San Diego, CA) and 12.5 nmol SYTOX Blue Dead Cell Stain (Invitrogen, Carlsbad, CA) 30 min prior to imaging. For the duration of experiments, mice were anesthetized by inhaled isoflurane according to standard procedures and body temperature was maintained with a 37 °C heated stage. Mice were placed in the supine position and the left lobe of the liver was exteriorized and treated with 1500 V/cm IRE. The exposed lobe was placed on saline-soaked gauze to prevent dehydration and the gauze was gently suspended ½ inch above the abdomen using optical posts and 90° angle post clamps (Thor Labs, Newton, NJ). The liver was further stabilized using a flanged vacuum window with an 8 mm coverslip set to 15–25 mmHg of suction (Amvex, ON, Canada). Intravital imaging was performed using a Nikon A1R upright laser scanning confocal microscope (Nikon Instruments, Melville, NY) equipped with a Mai Tai DeepSee IR laser (Spectra-Physics, Santa Clara, CA) tuned to 920 nm using a 25x/1.1 NA Plan Apo LWD water immersion objective (Nikon). Neutrophils were identified as eGFP^+^ cells, as confirmed by the neutrophil specific Ly6G-PE antibody (1A8, Biolegend, Bisbane, CA) given IV at a dose of 2 μg /mouse. Each mouse was imaged over a 3-h period at randomly selected regions within the injury area. Neutrophil movement and velocity were analyzed using Imaris 9.5 software (Oxford Instruments, Shanghai, China) and neutrophil number was quantified using the ImageJ Cell Counter pluggin (ImageJ, v1.36b)^68^.

### Isolation of liver myeloid cells

Myeloid cells were isolated from the liver by retrograde perfusion with HBSS (Gibco, Carlsbad, CA) containing 78.08 U/mL collagenase IV (Warthington, Newark, NJ) and 20 μg/ml DNAse I (Roche, Indianapolis, IN) for 6 min. Partially digested livers underwent a second digestion with 0.104 U/ml liberase (Roche) and 10 μg/ml DNAse I (Roche) for 30 min in a shaking 37 °C water bath, and were mechanically disrupted by passing through a 70 μM nylon cell strainer (BD Biosciences, Bedford, MA). Live myeloid cells were enriched from liver suspensions by density gradient centrifugation using a 40/60% Percoll (Millipore Sigma, St. Louis, MO) gradient. Cells were harvested at the gradient interface, washed, and then resuspended in PBS (Gibco) containing 0.5% bovine serum albumin and 2 mM EDTA.

### Flow cytometry

Cell suspensions were incubated with Ghost Dye BV510 Live/Dead stain (Tonbo Biosciences, San Diego, CA) at room temperature for 20 min, washed, incubated with 1:250 Fc block (2.4G2, BD Biosciences, Franklin Lakes, NJ) at 4 °C for 10 min, and then incubated with fluorochrome-labeled antibodies at 4C for 30 min using the following antibodies: CD19-PerCP/Cyanine5.5 (1:400; 6D5, Biolegend), CD64-PE/Cy7 (1:200; X54-5/7.1, Biolegend), Ly6G-PE (1:200; 1A8, Biolegend), CD11b-APC-780 (1:200; M1/70, eBioscience), CD45-AF700 (1:200; 30-F11, eBioscience), F4/80-APC (1:100; BM8, Biolegend), CD90.2-BV570 (1:100; 30-H12, Biolegend), Gr-1-BV711 (1:200; RB6-8C5, Biolegend) , CD11c-Bv650 (1:200; N418, Biolegend), Ly6c-Bv605 (1:100; HK1.4, Biolegend), MHC Class II-e450 (1:400; M5/114.15.2, eBioscience). Cells were analyzed using an LSRII flow cytometer (BD Biosciences) through the UCSF Liver Center Flow Cytometry Core Facility. Analysis was performed with FlowJo v.10.7.1 (Tree Star, Ashland, OR).

### Neutrophil depletion

For neutrophil specific depletion, C57BL/6 mice were administered 200 μg of anti-Ly6G antibody (1A8; Bio-X-Cell, West Lebanon, NH) by intraperitoneal (IP) injection 24 h before IRE treatment, at the time of IRE treatment (day 0), and then on days 2, 4 and 6. Control mice were administered 200 μg rat IgG2a isotype control antibody (2A3; Bio-X-Cell) on the same schedule. To increase the efficiency of neutrophil depletion, 200 μg of anti-rat IgGk (MAR18.5; Bio-X-Cell) was administered to all mice by IP injection on days 1 and 3. Circulating neutrophils in the blood, were monitored by flow cytometry on days 0, 1 and 3 to assess the binding of neutrophils to the anti-Ly6G antibody in vivo and the efficiency of neutrophil depletion. Neutrophils were identified as CD45^+^CD19^-^CD90^-^MHCII^lo^CD11b^hi^Ly6C^int^GR1^+^. GR1 was used to identify neutrophils because the GR1 antibody binds to a different epitope of Ly6G than the depletion antibody.

### Immunohistochemistry

Mouse livers were fixed in 10% formalin. Paraffin embedded samples were cut to 5 μm sections and stained by HistoWiz Inc. (Brooklyn, NY) on a Bond Rx autostainer (Leica Biosystems, Buffalo Grove, IL) with enzyme treatment (1:1000) and anti-F4/80 (BM8) or anti-SOX9 (EPR14335) using standard protocols. Bond Polymer Refine Detection (Leica Biosystems) was used according to manufacturer's instructions. Sections were counterstained with hematoxylin. Whole slide scanning was performed on an Aperio AT2 (Leica Biosystems), visualized using Aperio Image Scope software (Leica Biosystems), and digitally analyzed by ImagJ^68^.

### Statistical analysis

One-way or two-way ANOVA, with multiple correction post-hoc tests where appropriate (Tukey or Holm-Sidak), or two-tailed t-tests were performed with GraphPad Prism v.9.0.2 (GraphPad, La Jolla, CA).

### Ethics declaration

All animal experiments were approved by the Institutional Animal Care and Use Committee at University of California, San Francisco, approval number AN181725. Mice used in these studies were cared for in accordance to the National Institutes of Health “Guide for the Care and Use of Laboratory Animals” and ARRIVE guidelines.

## Supplementary Information

Supplementary Video 1.

Supplementary Video 2.

Supplementary Video 3.

Supplementary Video 4.

Supplementary Video 5.

Supplementary Video 6.

Supplementary Information 1.
