# Supplementary material for: Neutrophils are important for the development of pro-reparative macrophages after irreversible electroporation of the liver in mice
Source: Sci Rep. 2021 Jul 22;11:14986. doi: 10.1038/s41598-021-94016-8 (PMC8298444; doi:10.1038/s41598-021-94016-8)
Supplement: Supplementary file 7 — Supplementary Information 1. [file 41598_2021_94016_MOESM7_ESM.pdf]

Neutrophils are Important for the Development of Pro-Reparative Macrophages after Irreversible Electroporation of the Liver in Mice

Maya Lopez-Ichikawa[1], Ngan K. Vu[1], Amar Nijagal[1], Boris Rubinsky[2], and Tammy T. Chang[1]*

[1]Department of Surgery, University of California, San Francisco, CA 94143

[2]Department of Mechanical Engineering, University of California, Berkeley, CA 94720

*Corresponding Author:

Tammy T. Chang, MD, PhD
University of California, San Francisco
513 Parnassus Ave., HSW 1601
San Francisco, CA 94143
Tel: 415-476-6069
Email: tammy.chang@ucsf.edu

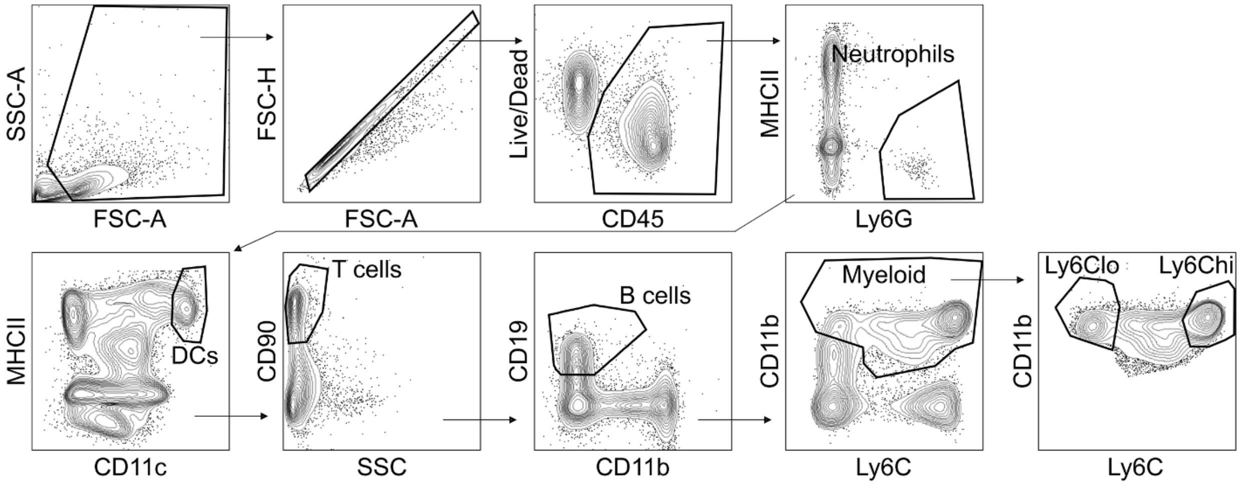

Supplemental Figure 1. Flow cytometry gating strategy for identification of liver myeloid cell populations.

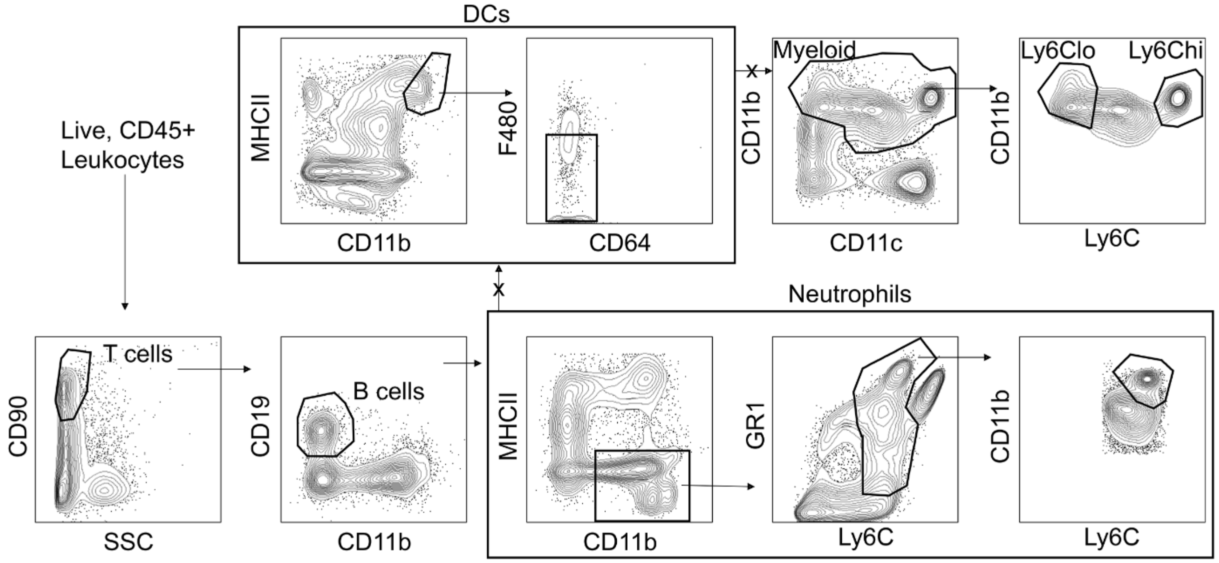

Supplemental Figure 2. Flow cytometry gating strategy for identification of liver myeloid cell populations after neutrophil depletion with anti-Ly6G antibody. The “x” represents excluded populations.

Supplemental Movie 1. Neutrophil movement (LysM-eGFP; green) in liver at baseline. PECAM-1 (red) staining shows microvasculature.

Supplemental Movie 2. Neutrophil movement (LysM-eGFP; green) in liver 30 minutes after IRE. PECAM-1 (red) staining shows microvasculature.

Supplemental Movie 3. Neutrophil movement (LysM-eGFP; green) in liver 1 hour after IRE. PECAM-1 (red) staining shows microvasculature.

Supplemental Movie 4. Neutrophil movement (LysM-eGFP; green) in liver 2 hours after IRE. PECAM-1 (red) staining shows microvasculature. Blue shows SYTOX.

Supplemental Movie 5. Neutrophil movement (LysM-eGFP; green) in liver 4 hours after IRE. PECAM-1 (red) staining shows microvasculature. Blue shows SYTOX.

Supplemental Movie 6. Liver microvascular remains patent and perfused 30 minutes after IRE. Green shows neutrophils (LysM-eGFP) and red shows Evan Blue dye flowing within vessels. The movie is sped up approximately 5x.
